# Editorial: Intelligent Conversational Agents

**DOI:** 10.3389/frai.2022.867834

**Published:** 2022-04-05

**Authors:** Marcus Specht, Catharine Oertel

**Affiliations:** ^1^Centre for Education and Learning, Delft University of Technology, Delft, Netherlands; ^2^Intelligent Systems, Delft University of Technology, Delft, Netherlands

**Keywords:** conversational agent(s), learning technologies, educational technology research, digital education, artificial intelligence in education

Conversational agent technologies embodied and conversational virtual agents are becoming more and more popular. Many people own an Alexa, Cortana or Echo or are talking to their virtual assistant on their phone. Indeed, such technologies have the potential of making our lives easier and relieving people from more repetitive tasks.

For example, it is imaginable that such systems are being used for financial applications by helping customers with frequently asked questions and advising them on more impactful decisions such as their pension plans in the long term. In fact, as a popular approach in customer service, conversational agents have found their way into educational applications. Yet, deploying such technologies in real-world settings poses many challenges. To motivate humans to engage in the longer term and/or repeated interaction with a conversational agent, they need to see a clear benefit. The provision of information but also the interaction itself play an important role. How to create a conversational memory and how to handle sensing in the wild still pose significant challenges.

The field is developing fast, and as the systematic literature review in this special issue highlights (Wollny et al.) chatbots have been used in various domains and taken different roles as assessor, mentor, coach, and others. The major objectives have been identified as skill improvement, efficiency of education, motivation, and accessibility of education. They are implementing classical schemes of educational technologies such as scaffolding, recommender systems or information agents. Also, the authors report that the potential of chatbots for personalized education has not yet been fully realized. There is a need for more research on applying learner modeling and adaptive feedback in the area. In conclusion, the authors also see the challenges of aligning the evaluation of chatbots systems with their implementation objectives, exploring more mentoring scenarios, and researching personalized and adaptive chatbots systems.

Addressing the issue of building more data-driven and flexible chatbots and especially acquiring the ethically balanced datasets for training these AI models in school contexts is the focus of the second article of Bailey et al.. The authors describe a sociocultural development approach to identify and validate relevant data sources in a child-centric perspective for training AI models. The authors discuss the complexity of questions when building on existing datasets and how building these datasets is related to consumerism. They give examples from the domains of children's books, children movies, and real-world interactions and explain how existing datasets could be used for training chatbots in these contexts. Especially in the setting with children, the authors stress the need for policymakers to facilitate the growth of AI models and their application in education.

Demonstrating the in-depth research needed for developing future chatbots, Mirzababaei and Pammer-Schindler, in their article, dive into the use of Toulmin's model for different types of assessment of conversational agents. Building on Toulmin's conceptual model of an argument, the authors work toward building an argumentation chatbot. In a first step, a classifier model to identify users' arguments, second for setting up a conditional dialogue structure and third also model different types of wrongness in the argumentation.

Finally, one of the enormous potentials conversational agents have is to support education at scale. Tegos et al. developed a prototype system called PeerTalk that uses a conversational agent to scaffold students collaboration in MOOCs. Their studies show encouraging findings in terms of system efficiency and usability levels.

Overall the contributions in the special issue show the broad range of domains and educational settings in which conversational agents have been adopted. Second, they stress the need to develop ethical datasets and link them to human learning processes and the pivotal development points. Third, they stress the need for a micro lens to research and create specific scenarios based on educational models.

## Author Contributions

All authors listed have made a substantial, direct, and intellectual contribution to the work and approved it for publication.

## Conflict of Interest

The authors declare that the research was conducted in the absence of any commercial or financial relationships that could be construed as a potential conflict of interest.

## Publisher's Note

All claims expressed in this article are solely those of the authors and do not necessarily represent those of their affiliated organizations, or those of the publisher, the editors and the reviewers. Any product that may be evaluated in this article, or claim that may be made by its manufacturer, is not guaranteed or endorsed by the publisher.

